# Heterogeneity of variance and genetic parameters for milk production in cattle, using Bayesian inference

**DOI:** 10.1371/journal.pone.0288257

**Published:** 2023-07-12

**Authors:** Raimundo Nonato Colares Camargo Júnior, Luane da Silva Fernandes, João Cláudio do Carmo Panetto, Marcos Vinicius Gualberto Barbosa da Silva, Cláudio Vieira de Araújo, André Guimarães Maciel e Silva, José Ribamar Felipe Marques, Welligton Conceição da Silva, Simone Inoe de Araújo, Sâmia Rubielle Silva de Castro, Lilian Kátia Ximenes Silva, Simone Vieira Castro, José de Brito Lourenço Júnior

**Affiliations:** 1 Postgraduate Program in Animal Science (PPGCAN), Institute of Veterinary Medicine, Federal University of Para (UFPA), UFRA, Brazilian Agricultural Research Corporation (EMBRAPA), Castanhal, PA, Brazil; 2 Federal University of Mato Grosso (UFMT), Sinop, Mato Grosso, Brazil; 3 Embrapa Gado de Leite, Juiz de Fora, Brazil; 4 Federal University of Pará (UFPA), Castanhal, Pará, Brazil; 5 Embrapa Amazônia Oriental, Belém, Pará, Brazil; 6 Harvard Center for Comparative Medicine, Boston, Massachusetts, United States of America; 7 Federal University of Pará (UFPA), Castanhal, Pará, Brazil; 8 Catholic University Center of Tocantins, Tocantins, TO, Brazil; Universidade Federal de Mato Grosso do Sul, BRAZIL

## Abstract

The goal of this study was to verify the effect of heterogeneity of variance (HV) on milk production in up to 305 days of lactation (L305) of daughters of Girolando, Gir and Holstein sires, as well as in the genetic evaluation of these sires and their progenies. in Brazil. The model included contemporary groups (consisting of herd, year and calving season) as a fixed effect, cow age at calving (linear and quadratic effects) and heterozygosity (linear effect) as covariates, in addition to the random effects of direct additive genetic and environmental, permanent and residual. The first analysis consisted of the single-trait animal model, with L305 records (disregarding HV). The second considered classes of standard deviations (SD): two-trait model including low and high classes (considering HV), according to the standardized means of L305 for herd-year of calving. The low SD class was composed of herds with SD equal to or less than zero and the high class with positive SD values. Estimates of (co)variance components and breeding values were obtained separately for each scenario using Bayesian inference via Gibbs sampling. Different heritability was estimated. Higher for the high DP class in the Gir (0.20) and Holstein (0.15) breeds, not occurring the same in the Girolando breed, with a lower value among the classes for the high DP (0.10). High values of genetic correlations were also found between low and high SD classes (0.88; 0.85 and 0.79) for the Girolando, Gir and Holstein breeds, respectively. Like the order correlations (Spearman) which were also high for the three breeds analyzed (equal to or above 0.92). Thus, the presence of HV had a smaller impact for L305 and did not affect the genetic evaluation of sires.

## Introduction

The use of local breeds adapted to a specific environment is one of the OIE’s recommendations [1], seeking to improve animal health conditions, as well as promote the sustainability of production systems. In addition, to improve the productive characteristics of dairy cattle, changes have been made in the management of herds (whether nutritional, ambience and/or reproductive) and/or the use of breeding strategies [2–4]. The genetic progress of herds has become increasingly widespread with the intense use of reproductive biotechnologies, resulting in the distribution of genetic material of animals, mainly sires, with high productive potential in different environments and contexts of dairy production around the world [5].

In this context, genotype x environment interaction (GEI) emerges as a possible problem in defining strategies for the use of genetic resources, due to the fact that genotypes with favorable performance in one environment may not have all their genetic potential expressed in another environment [6]. In addition, there is a tendency for GEI to be ignored in genetic evaluations, due to the low accuracy in selecting for performance in extreme environments, when there is limited information to estimate the genetic value and due to the complexity of the models used [7]. As a result, we can select some breeders in the wrong way, with better performances depending on the breeding environment provided to them and not by their genetic potential [8, 9].

The heterogeneity of variance in milk production is a form of GEI and when it occurs, the production of the progenies of a given breeder will be weighted by the proportion of SD of the herds in which their progenies are being evaluated. The result of this is that the productions of these daughters, from more variable herds, will present a greater weight in the genetic evaluation of breeders than progenies with production from less variable herds [10].

However, if the GEI is not expressive, records of daughters in different environments may alleviate the lower response to selection caused by GEI. If there is significant interaction between genotype and environment in the expression of a given productive characteristic, better said, if the genetic correlation between environments is less than 0.70; different improvement programs will be required for genetic assessment in each of the environments [11].

Therefore, it is essentially important to choose appropriate environmental descriptors in GEI studies [12–14]. The average level of production of each herd, for a given characteristic, is constantly used as an indicator of the effect of the environment to which the progeny is inserted, since this is an easily accessible information that associates numerous other environmental factors that influence the expression of the phenotype [15–18].

India is the world’s largest dairy producer, with the largest proportion of production coming from small herds, averaging less than two dairy cows per herd. These cows are mostly multi-generation indefinite crosses between exotic dairy breeds and indigenous cattle, with no record of performance or pedigree. Initial results from a performance recording program indicate a strong potential for genomic selection to improve milk production in these animals. The performance of animals with different racial compositions, in different Indian environments, will allow better guidance for small producers on the ideal racial composition for each environment. These results highlight the effect of GEI on dairy production, showing that the best animals for one environment will not necessarily be the same in another [19].

Other authors also found the existence of the effect of GEI in several breeds, regions, countries, and levels of herd production and demonstrated that there is an *effect on the ranking* of breeders [20–22]. These results show the importance of considering GEI in genetic evaluations of dairy herds.

Therefore, the goal of this study was to evaluate the presence of variance heterogeneity for milk production up to 305 days of lactation in the various Brazilian herds of the Girolando, Gir and Holstein breeds, as well as to measure how much this heterogeneity can interfere in the classification of sires through genetic values.

## Materials and methods

### Animals and data consistency

**Fig 1:**
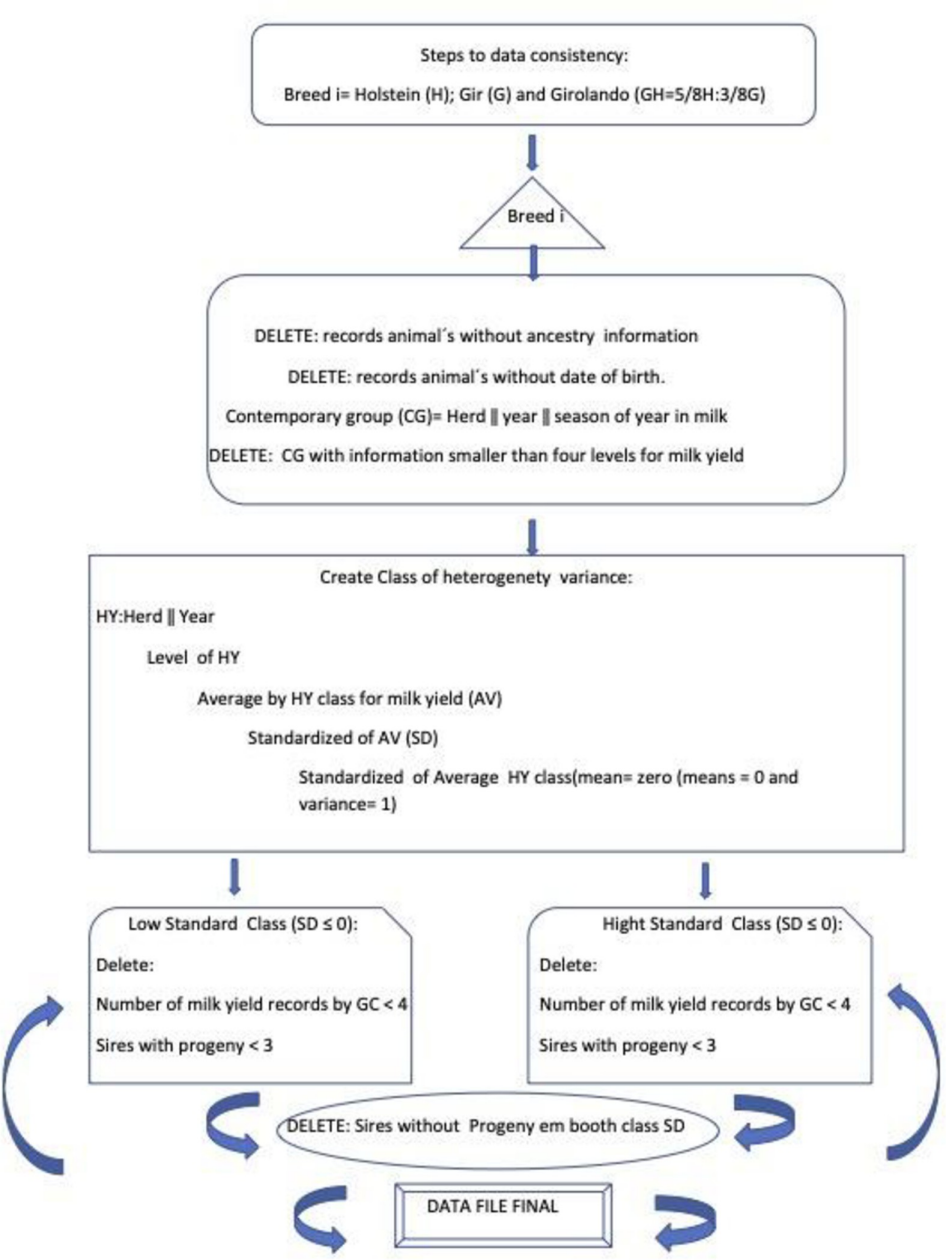
Iconic representation of steps to data consistency.

For the present study, information on sires and cows of gir, Girolando and Holstein breeds was used. Data were collected from the Programa de Melhoramento Genético da Raça Girolando (PMGG), under the technical coordination of the Embrapa Gado de Leite (Fig 1).

Phenotype files contained 10,482, 16,439 and 65,235 records of milk production within 305 days of lactation of cows (Girolando) daughters of 146, 189 and 873 breeding animals of the Girolando breed (5/8 Holstein), Gir and Holstein, respectively, between 1994 and 2019.

To perform the analyses, we chose to separate the daughters to be evaluated, according to the race of their parent, performing the correction of direct heterozygosis (DH), according to the equation proposed by [23]:

$$DH=a_{i}^{t}\;a_{j}^{v}+a_{j}^{t}\;a_{i}^{v}$$

Where, $a_{i}^{t}$ e $a_{i}^{v}$ denote the proportion of the breed ‘i’ in the sires and cow (mother) of the progeny.

For data consistency, proles records were eliminated without ancestry information, no date of birth or date of birth. The total milk yield (MY), in each breed, was standardized for 305 days in lactation (MY305) through the expression:

MY305=MY+b1(LL‐305)

Were, LL represents the length of lactation and b1 is the linear regression coefficient of MY as a function of LL. Milk production was limited up to 305 days at a maximum of up to 25,000 liters/lactation, with the age of the cow at delivery of up to 120 months.

Observations regarding herd-year-season classes of delivery with several information smaller than four were excluded. To ensure the genetic connection between the Classes of SD, breeders should have at least two daughters in each class and in both classes.

The months in which the deliveries occurred were grouped into two seasons, the dry season occurring from April to September and rain from October to March. These seasons were included in the formation group of contemporaries by the junction of the herd classes, year, and parting season.

### Statistical model used to estimate genetic parameters

To perform the analysis of the heterogeneity of variance among the herds, the data were analyzed in two situations. In the first, disregarding different variances of milk production among the herds-year of delivery (general analysis), obtaining estimates of the components of (co)variance and prediction of the genetic values of individuals for milk production.

In the second approach, milk production was considered in each phenotypic SD class as a distinct characteristic, obtaining estimates of the components of (co)variance and prediction of the genetic values of the individuals in each class.

The evaluation model included the fixed effects of contemporary group (composed of herd-year-season of delivery), the age of the cow at delivery (linear and quadratic effects) and degree of heterozygosis (linear effect) as covariates, and additive genetic effects of animal, permanent and residual environment as random effects.

### Model that disregards variance heterogeneity

In the general analysis, to obtain the estimates of the variance components, as well as the genetic values of the animals, the following animal model was used:

y=Xb+Za+Wp+e

Where, **y** is the vector of n milk production observations; **b** is the fixed effects vector; **a** is the vector of genetic values of the animals; **p** is a vector of values referring to the permanent effect of the environment on the animals; **e** is a vector of residuals of the same dimension as **y**. **X**, **Z**, **W** are the corresponding incidence matrices of fixed, additive genetic and permanent environment effects as random effects, respectively.

### Model considering variance heterogeneity

Classes of herd-year of calving were defined and, subsequently, the milk production averages were calculated in each of these classes. The mean values of the classes were standardized (considering mean equal to zero (*μ*_*c*_ = 0) and variance equal to one (*σ*_*c*_ = 1)) and then used to define the SD classes.

The low SD class consisted of standardized mean values equal to and less than zero. Otherwise, the high SD class was constituted with positive standardized mean values. In this way, it was possible to separate the herds that were on opposite sides of the normal data distribution curve. The following formula was used to standardize the mean:

$$Z=\frac{\left(X-\mu \right)}{\sigma }$$

Where, Z = standardized mean; X = mean milk production of herd-year of calving classes; μ = general average of production up to 305 days; σ = general standard deviation of production up to 305 days.

In this analysis, which considers each class of SD as a distinct trait, obtaining variance components and genetic values of breeders for milk production, taking into account a joint distribution of traits, considered the following model:

$$y_{i}=X_{i}b_{i}+Z_{i}a_{i}+W_{i}p_{i}+e$$

Where, yi = milk production in the i-th SD class and the other terms that represent the milk production in each SD class are the same previously described in the single trait model. For the components of (co)variances, distributions were assumed, the Inverse-Wishart Conjugate Prior.

The analyzes were performed by Bayesian inference using the BLUPF90 family programs [24]. A total of 300,000 Gibbs samples were generated, considering an initial discard period of 30,000 iterations. To minimize the effects of the initial values and to ensure the independence of the samples, a sampling interval of 10 iterations was considered, generating a total of 27,000 samples of the estimates of the variance components. The convergence criterion used was the Geweke diagnosis [25].

### Correlation between genetic values

Spearman correlation coefficients were calculated for all combinations of estimated genetic value sets and also considering only the top 20% sires and progenies (cows) based on the genetic value in general analysis, of the different models, within each breed. In this way, it is possible to verify if there was a reranking of the animals, due to the heterogeneity of variance.

The accuracy (*r*) of the estimated breeding values was obtained using the following formula, according to [26]:

$$r=\sqrt{1+\frac{PEV}{\sigma_{a}^{2}}}$$

Where PEV is the variance of the prediction error and $\sigma_{g}^{2}$ is the additive genetic variance of the trait.

The consistency of the database, as well as spearman’s genetic and correlations, were performed through the Statistical Program SAS [27].

### Results and discussion

Subsequent values observed for means, standard deviations, coefficients of variation (CV), number of lactations and number of group of contemporaries, for milk production, in each phenotypic SD class and in general analysis are presented in Table 1 for the Girolando, Gir and Holstein breeds.

**Table 1:** Descriptive data statistics including mean (kg), standard deviation (SD, kg), coefficient of variation (CV, %), number of lactations (NL) and number of contemporaneous groups (CG).

		General Analysis	Standard deviation classes	
			Low	High
Girolando	Mean (Kg)	4065.10	2883.56	5122.75
	SD (Kg)	2080.15	1463.95	1978.36
	CV (%)	51.17	50.77	38.62
	NL	10482	4951	5531
	CG	1862	915	947
Gir	Mean (Kg)	5063.16	3542.61	6340.51
	SD (Kg)	2674.01	1992.54	2499.64
	CV (%)	52.81	56.25	39.42
	NL	16439	7505	8934
	CG	2846	1477	1369
Holstein	Mean (Kg)	5247.84	3822.28	6392.23
	SD (Kg)	2576.54	1954.08	2441.63
	CV (%)	49.10	51.12	38.20
	NL	65235	29049	36186
	CG	5551	2746	2805

The CV were high, mainly in the herds included in the low SD class, reflecting, especially, in inequality of productions between the studied herds.

The herds included in the high SD class had a higher mean milk production for all breeds studied. However, the highest SD was found in the general analysis, with no increase in the mean associated with an increase in SD in the three breeds analyzed.

A similar result was observed by [28] and [29], who also found that increasing the mean did not necessarily increase the standard deviation. Contrary to what was found by [30, 31], when working with the Brown-Swiss breed, where an association between mean and SD within the herd was verified.

It was possible to observe that the high SD class with the highest average, highest SD and lowest CV, between classes, exhibited homogeneity in the milk production of the animals, in the three breeds used in this study, indicating that the criterion for forming the SD classes was efficient.

In all breeds, there was an increase in the posterior means of the estimates of the variance components referring to the additive genetic effect, permanent environment and residual effect (Tables 2–4).

**Table 2:** Means, respective SD and credibility interval (HPD = 95%) for posterior estimates of variances, heritability (h^2^), repeatability (rep) and genetic correlation (r_g_) for milk production up to 305 days of daughters of sires of the Girolando breed, in general analysis and in the different classes of phenotypic SD.

	General analysis	SD classes	
		Low	High
σ^2^a	2114.34 ± 687.17	2077.75 ± 597.03	2979.68 ± 1010.68
HPD	(862.10–3483.0)	(959.70–3239.0)	(1122.0–4967.0)
σ^2^p	5465.80 ± 658.71	3507.01 ± 564.33	6956.49 ± 994.52
HPD	(4186.0–6759.0)	(2467.0–4651.0)	(5064.0–8974.0)
σ^2^e	16927.77 ± 379.23	11711.42 ± 406.76	21192.93 ± 661.77
HPD	(16190.0–17670.0)	(10940.0–12530.0)	(19950.0–22520.0)
h^2^	0.09 ± 0.03	0.12 ± 0.03	0.10 ± 0.03
Rep	0.31 ± 0.02	0.32 ± 0.02	0.32 ± 0.02
r_g_	-	0.88 ± 0.10	

^a^σ^2^a: additive genetic variance; ^b^σ^2^p: permanent environment variance; ^c^σ^2^e: residual variance.

**Table 3:** Means, respective SD and credibility interval (HPD = 95%) for posterior estimates of variances, heritability (h^2^), repeatability (rep) and genetic correlation (r_g_) for milk production up to 305 days of daughters of sires of the Gir breed, in general analysis and in the different classes of phenotypic SD.

	General analysis	SD classes	
		Low	High
σ^2^a	6958.15 ± 1059.96	5870.29 ± 1240.74	9942.05 ± 1777.91
HPD	(4919.0–8969.0)	(3554.0–8543.0)	(6637.0–13460.0)
σ^2^p	7829.34 ± 907.10	6942.32 ± 1243.21	8262.16 ± 1547.73
HPD	(6098.0–9585.0)	(4462.0–9303.0)	(5342.0–11290.0)
σ^2^e	26867.49 ± 496.20	19476.60 ± 724.47	30988.95 ± 828.94
HPD	(25910.0–2784.0)	(18080.0–20920.0)	(29400.0–32650.0)
h^2^	0.17 ± 0.02	0.18 ± 0.04	0.20 ± 0.03
Rep	0.35 ± 0.02	0.40 ± 0.02	0.37 ± 0.18
r_g_	-	0.85 ± 0.08	

^a^σ^2^a: additive genetic variance; ^b^σ^2^p: permanent environment variance; ^c^σ^2^e: residual variance.

**Table 4:** Means, respective SD and credibility interval (HPD = 95%) for posterior estimates of variances, heritability (h^2^), repeatability (rep) and genetic correlation (r_g_) for milk production up to 305 days of daughters of sires of the Holstein breed, in general analysis and in the different classes of phenotypic SD.

	General analysis	SD classes	
		Low	High
σ^2^a	4993.91 ± 399.15	3393.68 ± 428.97	7202.86 ± 669.20
HPD	(4235.0–5758.0)	(2590.0–4207.0)	(5926.0–8512.0)
σ^2^p	6522.89 ± 380.99	6005.77 ± 457.96	6737.18 ± 655.87
HPD	(5770.0–7263.0)	(5124.0–6878.0)	(5424.0–8035.0)
σ^2^e	29162.51 ± 254.63	21319.34 ± 316.36	34589.79 ± 443.27
HPD	(28660.0–29650.0)	(20740.0–21990.0)	(33710.0–35440.0)
h^2^	0.12 ± 0.01	0.11 ± 0.01	0.15 ± 0.01
Rep	0.28 ± 0.01	0.31 ± 0.01	0.29 ± 0.01
r_g_	-	0.79 ± 0.06	

^a^σ^2^a: additive genetic variance; ^b^σ^2^p: permanent environment variance; ^c^σ^2^e: residual variance.

This increase occurred from low to high phenotypic SD, however, this change was not proportional to the point that heritabilities, repeatability and correlations remained constant in the different analyses.

The increase in the estimates of the genetic and residual variance components, according to the increase in the production level of the herds, has been reported in other studies [32–35] with dairy cattle, using approaches different from the one used in this study.

It is noteworthy that, for the Girolando breed, the posterior mean of the estimation of permanent environment variance for the high SD class was 98.36% higher than the low SD class. This had a great impact on the calculation of the total phenotypic variance. In general, environmental variation was responsible for an important part of the total variation of the evaluated trait, causing low heritabilities.

Geweke’s criterion [25], which consists of indicating the convergence of the mean, resulted in a probability value associated with the test always greater than the 5% level of significance adopted, indicating convergence of the chains and the validity of the Bayesian analysis.

For the Girolando breed, the posterior means of heritability, considering the SD classes, were close, being 0.10 for the high SD and 0.12 for the low SD. This shows that, although the herds are in different environments, genetic factors act in a similar way in both.

Comparing the a posteriori mean of heritability in general analysis (0.09), there is a great difference in relation to the values reported by [36], who obtained heritability of up to 0.1, much larger than that found here. This difference may be due to the elimination of short lactations, less than 120 days, which caused a decrease in the range of data in that study. Another point could be the fact that they used blood degrees of the Girolando breed for the cows and not according to the breed of the sire, as performed in the present study.

The subsequent mean of heritability found in the general analysis (0.17) for the Gir breed was much lower than that reported by [37] with values up to 0.39. Furthermore, in our study, the high SD class (0.18) was only 10% higher than the heritability estimate for the low SD class (0.20).

Posterior means of heritability of 0.12, 0.15 and 0.11 (general analysis, high and low SD, respectively) approximate from those found by [38] in general analysis (0.14) and different by [39] in general analysis (0.18), for Holstein animals.

Heritability values were, in general, higher in the high SD classes, with the exception of the Girolando breed, mainly due to the influence of the posterior estimate of the permanent environment variance in this breed.

In many cases, the more intensive the management of the herd for milk production, the greater the heritability [40] and this can be explained by the greater opportunity for cows to express their genetic potential in herds with high levels of production, due to the better environment offered, which is characterized by greater control of the health aspects of the cows and better feeding [41–43].

Differences in heritability estimates may also be due to non-compliance between the populations studied and the different analysis methods used. However, ignoring these differences between heritability estimates, although small, raises doubts about the reliability of estimated reproductive values.

In addition, heritability, and gradients of genetic variation between environments could imply a higher genetic response in herds with more intensive management. It becomes evident the need for further research considering VH in genetic evaluation models, including through models of reaction norms.

Repeatability values indicated reasonable similarity between consecutive lactations of the same animal, suggesting the importance of permanent environmental effects in determining the expression of this trait. In general, comparing within each breed, repeatability values did not differ greatly, ranging from 0.31 to 0.32 (Girolando), 0.35 to 0.40 (Gir) and 0.28 to 0.31 (Holstein).

Animals of the Girolando breed reported a repeatability value equal to 0.93 in general analysis The animals of the Girolando breed have repeatability values ranging from 0.33 [44] to 0.93 [45] in the general analysis, much higher than that found in this study (0.31) for the same breed. A large part of this difference is probably due to our lower heritability values found. In other words, genetic variability contributed to a lesser extent in the repeatability estimates [46].

For the Girolando, Gir and Holstein breeds, high values of genetic correlations were found (Tables 2–4), between low and high SD classes (0.88; 0.85 and 0.79). Studies on GEI [47, 48] concluded that when there is a genetic correlation greater than 0.61 between two environments, a higher average genetic gain is achieved for all sires in both environments.

In view of this, as reported by other authors [30, 49–51], our results indicate a significant absence of variance heterogeneity for milk production up to 305 days in the analyzed herds, that is, it is expected that the genetic value and the ranking of the sires are the same in the two SD classes.

The order correlations (Spearman) between the genetic values obtained in the low and high SD analyzes and those obtained in the general analysis, taking into account all sires, were equal to or greater than 0.92 (Table 5), and were shown to be significant (p<0.01) for the three breeds studied.

**Table 5:** Spearman correlation estimates (above the diagonal) between all sires in general analysis and in each phenotypic SD class and, for the top 20% sires (below the diagonal); classified according to the genetic value in general analysis, for milk production up to 305 days, for the daughters of Girolando, Gir and Holstein breeders.

	Girolando			Gir			Holstein		
	General	Low	High	General	Low	High	General	Low	High
General	-	0.95	0.99	-	0.96	0.99	-	0.97	0.98
Low	0.86	-	0.95	0.76	-	0.93	0.82	-	0.92
High	0.97	0.90	-	0.95	0.63	-	0.93	0.65	-

The order correlations (Spearman) between the genetic values obtained in the low and high SD analyzes and those obtained in the general analysis, taking into account all sires, were equal to or greater than 0.92 (Table 5), and were shown to be significant (p<0.01) for the three breeds studied.

However, when the correlation is performed for the 20% of the best sires, classified based on the genetic values estimated in the general analysis, there were reductions in the order correlations. Becoming higher for Gir and Holstein breeds, between low and high SD classes, 32.26% and 29.35%, respectively. As for the Girolando breed, the greatest impact was between the general analysis and the low SD class, with a reduction of this correlation by 9.47%.

The greater the selection intensity, the lower the percentage of common animals between the classification groups, since the effect of errors in the classification order is more evident when a small number of animals are selected.

The greater association of high SD classes with the general analysis indicates that more variable herds contribute with greater participation in the prediction of genetic values, in a situation that disregards variance heterogeneity. This is because the high SD classes had greater additive genetic variability.

As with the sires, the order correlation was performed for their progenies (Table 6). Correlation coefficients considering all cows, according to sire breed (father), were also equal to or greater than 0.92. This means that their ranking remained similar between the different SD classes.

**Table 6:** Spearman correlation estimates (above diagonal) among all cows (daughters of Gir, Girolando, and Holstein sires), in overall analysis and in each phenotypic standard deviation class, and for the top 20% cows (below diagonal), ranked according to the genetic value in general analysis, for milk production up to 305 days.

	Girolando			Gir			Holstein		
	General	Low	High	General	Low	High	General	Low	High
General	-	0.96	0.99	-	0.95	0.99	-	0.98	0.99
Low	0.78	-	0.95	0.83	-	0.92	081	-	0.95
High	0.97	0.83	-	0.93	0.70	-	0.94	0.62	-

However, there was a downward trend in correlation values as selection pressure increased. This became evident when the Spearman correlation considering only 20% of the best cows, according to the genetic value in the general analysis, decreased to values up to 0.62. However, there was a downward trend in the correlation values, as there was an increase in the selective intensity of the sires, since the Spearman correlation considering only 20% of the best cows, according to the genetic value in the general analysis, decreased to values of up to 0.62.

A study analyzing breeding strategies in two environments, based on the highest mean genetic gain [52–56], found that the analysis was relatively insensitive to heritability, number of progenies per sire and the relative importance of both environments, but was very sensitive to intensity of selection, as occurred in this study. Another study [57] evaluating the weight of male and female cattle, but using linear regression, showed that males had higher body weights and greater gains in body mass (BW) compared to females, with advancing age.

Although alterations were observed in order correlations, it is verified in Table 7 that there was no drastic change in the number of animals, for sires and cows, which remained in the list of the ten best classified.

**Table 7:** Number of sires and their progeny (cows), in the top 10 classification with the model under general analysis that remain in the top 10 classification, with the analysis of the herds in the different classes.

	Girolando			Gir			Holstein		
	GA^a^	L^b^	H^c^	GA	L	H	GA	L	H
Sires	10	8	9	10	8	9	10	6	10
Cows	10	9	9	10	5	8	10	7	8

^a^GA: general analysis; ^b^L: low SD class; ^c^H: high SD class.

There were only two cases of considerable change. The first was in the Holstein breed, in which only 6 animals of the 10 bests, remain in the analysis, considering the low SD class (analysis of the sires). The second happened in the analysis of the cows, in which only 5 animals, out of the top 10, remain in the analysis considering the low SD class, in the Gir breed.

Other studies [2, 22, 58–63] show small GEI effects and correlation values close to unity, with no significant reclassification of sires for milk production, whether with reaction norms or with analysis of genetic and order correlations between environments.

This indicates that in this research herd environments (included in SD classes) may be similar in some respects, although they differed in herd size and location regions.

The HV existing in this study did not cause great harm in the genetic evaluation of sires in the three studied breeds. This was evidenced when observing the results obtained in the general analysis, which would similarly select the same sires within the HV classes.

## Conclusion

Estimates of heritability and genetic correlation for milk yield revealed that the heterogeneity of variance present is mainly genetic in nature. Therefore, the presence of variance heterogeneity did not affect the genetic evaluation of sires for the three breeds studied.

Spearman’s correlations showed that there was no difference in the classification of sires without considering HV. However, this pattern was altered when the selection intensity increased in the sires, resulting in a considerable change in their ranking.

In animal genetic evaluation models, it is important to verify the presence of HV, as well as its effect on the genetic evaluation of the breeders, especially in countries where there are large variations in the environment.

## Supporting information

S1 File (PDF)

## References

[1] OIE. Introduction to the Recommendations for Animal Welfare Article 2022 [updated 2023/04/28; cited 2023]. Available from: https://www.woah.org/fileadmin/Home/eng/Health_standards/tahc/current/chapitre_aw_introduction.pdf.

[2] Cao L, Liu H, Mulder HA, Henryon M, Thomasen JR, Kargo M, et al. Genomic Breeding Programs Realize Larger Benefits by Cooperation in the Presence of Genotype × Environment Interaction Than Conventional Breeding Programs. Frontiers in Genetics. 2020;11. doi: 10.3389/fgene.2020.00251 32373152 PMC7186425

[3] Silva WCd Silva JARd, Camargo-Júnior RNC Silva ÉBRd, Santos MRPd Viana RB, et al. Animal welfare and effects of per-female stress on male and cattle reproduction—A review. Frontiers in Veterinary Science. 2023;10. doi: 10.3389/fvets.2023.1083469 37065229 PMC10102491

[4] Silva WCd Silva ÉBRd, Santos MRPd Camargo Junior RNC, Barbosa AVC Silva JARd, et al. Behavior and thermal comfort of light and dark coat dairy cows in the Eastern Amazon. Frontiers in Veterinary Science. 2022;9. doi: 10.3389/fvets.2022.1006093 36187817 PMC9516290

[5] Moreira RP, Pinto LFB, Valloto AA, Pedrosa VB. Evaluation of genotype by environment interactions on milk production traits of Holstein cows in southern Brazil. Asian-Australas J Anim Sci. 2019;32(4):459–66. Epub 2018/07/31. doi: 10.5713/ajas.18.0174 ; PubMed Central PMCID: PMC6409447. 30056654 PMC6409447

[6] Cheruiyot EK, Nguyen TTT, Haile-Mariam M, Cocks BG, Abdelsayed M, Pryce JE. Genotype-by-environment (temperature-humidity) interaction of milk production traits in Australian Holstein cattle. Journal of Dairy Science. 2020;103(3):2460–76. doi: 10.3168/jds.2019-17609 31864748

[7] Tiezzi F, Maltecca C. Genotype by Environment Interactions in Livestock Farming. In: Meyers RA, editor. Encyclopedia of Sustainability Science and Technology; doi: 10.1007/978-1-4939-2493-6_1115–1 New York, NY: Springer New York; 2020. p. 1–21.

[8] Araújo CVd Resende GSA, Araújo SI Rennó FP, Tomazini API Marques JRF. Interação genótipo x ambiente para produção de leite na raça Pardo Suíço, utilizando-se inferência Bayesiana. Acta Scientiarum Animal Sciences. 2009;31(2):205–11. doi: 10.4025/actascianimsci.v31i2.5197

[9] Araujo CVd Oliveira LdA, Araujo SI Silva DAd, Silva AAd. Impacto da distribuição de número desigual de progênies por reprodutor na avaliação genética de animais, em ambientes com presença de heterogeneidade de variância ambiental. Ciência Animal Brasileira. 2017;18. doi: 10.1590/1089-6891v18e-16286

[10] Silva DA, Lopes PS, Costa CN, Silva AA, Silva HT, Silva FF, et al. Genotype by environment interaction for Holstein cattle populations using autoregressive and within- and across-country multi-trait reaction norms test-day models. animal. 2021;15(2):100084. doi: 10.1016/j.animal.2020.100084 33712214

[11] Buaban S, Puangdee S, Duangjinda M, Boonkum W. Estimation of genetic parameters and trends for production traits of dairy cattle in Thailand using a multiple-trait multiple-lactation test day model. Asian-Australas J Anim Sci. 2020;33(9):1387–99. Epub 2020/02/15. doi: 10.5713/ajas.19.0141 ; PubMed Central PMCID: PMC7468173. 32054206 PMC7468173

[12] König S, May K. Invited review: Phenotyping strategies and quantitative-genetic background of resistance, tolerance and resilience associated traits in dairy cattle. Animal. 2019;13(5):897–908. Epub 2018/12/07. doi: 10.1017/S1751731118003208 30523776

[13] Schmid M, Bennewitz J. Invited review: Genome-wide association analysis for quantitative traits in livestock–a selective review of statistical models and experimental designs. Arch Anim Breed. 2017;60(3):335–46. doi: 10.5194/aab-60-335-2017

[14] Bennewitz J, Edel C, Fries R, Meuwissen THE, Wellmann R. Application of a Bayesian dominance model improves power in quantitative trait genome-wide association analysis. Genetics Selection Evolution. 2017;49(1):7. doi: 10.1186/s12711-017-0284-7 28088170 PMC5237573

[15] Toro-Ospina AM, Faria RA, Dominguez-Castaño P, Santana ML, Gonzalez LG, Espasandin AC, et al. Genotype–environment interaction for milk production of Gyr cattle in Brazil and Colombia. Genes & Genomics. 2023;45(2):135–43. doi: 10.1007/s13258-022-01273-6 35689753

[16] Clasen JB, Fogh A, Kargo M. Differences between performance of F1 crossbreds and Holsteins at different production levels. Journal of Dairy Science. 2019;102(1):436–41. doi: 10.3168/jds.2018-14975 30415848

[17] Edwards J. Comparison of milk production and herd characteristics in New Zealand herds milked once or twice a day. Animal Production Science. 2019;59(3):570–80. doi: 10.1071/AN17484

[18] Ramírez-Rivera EJ, Rodríguez-Miranda J, Huerta-Mora IR, Cárdenas-Cágal A, Juárez-Barrientos JM. Tropical milk production systems and milk quality: a review. Tropical Animal Health and Production. 2019;51(6):1295–305. doi: 10.1007/s11250-019-01922-1 31134554

[19] Al Kalaldeh M, Swaminathan M, Gaundare Y, Joshi S, Aliloo H, Strucken EM, et al. Genomic evaluation of milk yield in a smallholder crossbred dairy production system in India. Genetics Selection Evolution. 2021;53(1):73. doi: 10.1186/s12711-021-00667-6 34507523 PMC8431883

[20] Rodríguez-Bermúdez R, Miranda M, Baudracco J, Fouz R, Pereira V, López-Alonso M. Breeding for organic dairy farming: what types of cows are needed? Journal of Dairy Research. 2019;86(1):3–12. Epub 2019/03/25. doi: 10.1017/S0022029919000141 30907720

[21] Berry DP. Invited review: Beef-on-dairy—The generation of crossbred beef × dairy cattle. Journal of Dairy Science. 2021;104(4):3789–819. doi: 10.3168/jds.2020-19519 33663845

[22] Santos JC, Malhado CHM, Cobuci JA, de Rezende MPG, Carneiro PLS. Genotype-environment interaction for productive traits of Holstein cows in Brazil described by reaction norms. Tropical Animal Health and Production. 2020;52(5):2425–32. doi: 10.1007/s11250-020-02269-8 32297042

[23] Wolf J, Distl O, Hyánek J, Grosshans T, Seeland G. Crossbreeding in farm animals. V. Analysis of crossbreeding plans with secondary crossbred generations. Journal of Animal Breeding and Genetics. 1995;112(1–6):81–94. doi: 10.1111/j.1439-0388.1995.tb00545.x

[24] Misztal I, Tsuruta S, Strabel T, Auvray B, Druet T, Lee D, editors. BLUPF90 and related programs (BGF90). Proceedings of the 7th world congress on genetics applied to livestock production; 2002: Montpellier.

[25] Geweke JF. Evaluating the accuracy of sampling-based approaches to the calculation of posterior moments. Oxford: Federal Reserve Bank of Minneapolis, 1991.

[26] Fernandes Júnior GA, Rosa GJM, Valente BD, Carvalheiro R, Baldi F, Garcia DA, et al. Genomic prediction of breeding values for carcass traits in Nellore cattle. Genetics Selection Evolution. 2016;48(1):7. doi: 10.1186/s12711-016-0188-y 26830208 PMC4734869

[27] Institute S. SAS/ETS 9.1 User’s Guide: SAS Institute; 2004.

[28] Araujo CVd Oliveira LdA, Araujo SI Silva DAd, Silva AAd. Impacto da distribuição de número desigual de progênies por reprodutor na avaliação genética de animais, em ambientes com presença de heterogeneidade de variância ambiental. Ciência Animal Brasileira. 2017;18:1–9. doi: 10.1590/1089-6891v18e-16286

[29] Santos JC. INTERAÇÃO GENÓTIPOS AMBIENTES EM CARACTERÍSTICAS PRODUTIVAS E REPRODUTIVAS DE VACAS HOLANDESAS VIA NORMAS DE REAÇÃO 2018.

[30] Araújo CVd Torres RdA, Rennó FP Pereira JC, Torres Filho RdA Araújo SI, et al. Heterogeneidade de Variância na Avaliação Genética de Reprodutores da Raça Pardo-Suíça no Brasil. Revista Brasileira de Zootecnia. 2002;31. doi: 10.1590/S1516-35982002000600004

[31] Schmid M, Imort-Just A, Emmerling R, Fuerst C, Hamann H, Bennewitz J. Genotype-by-environment interactions at the trait level and total merit index level for milk production and functional traits in Brown Swiss cattle. Animal. 2021;15(1):100052. doi: 10.1016/j.animal.2020.100052 33516040

[32] Rowiński PK, Rogell B. Environmental stress correlates with increases in both genetic and residual variances: A meta‐analysis of animal studies. Evolution. 2017;71(5):1339–51. doi: 10.1111/evo.13201 28186615

[33] Yao C, de los Campos G, VandeHaar MJ, Spurlock DM, Armentano LE, Coffey M, et al. Use of genotype × environment interaction model to accommodate genetic heterogeneity for residual feed intake, dry matter intake, net energy in milk, and metabolic body weight in dairy cattle. Journal of Dairy Science. 2017;100(3):2007–16. doi: 10.3168/jds.2016-11606 28109605

[34] Lee S, Do C, Choy Y, Dang C, Mahboob A, Cho K. Estimation of the genetic milk yield parameters of Holstein cattle under heat stress in South Korea. Asian-Australasian Journal of Animal Sciences. 2019;32(3):334–40. Epub 2018/07/31. doi: 10.5713/ajas.18.0258 ; PubMed Central PMCID: PMC6409471. 30056660 PMC6409471

[35] Scarso S, McParland S, Visentin G, Berry DP, McDermott A, De Marchi M. Genetic and nongenetic factors associated with milk color in dairy cows. Journal of Dairy Science. 2017;100(9):7345–61. doi: 10.3168/jds.2016-11683 28711262

[36] Radjabalizadeh K, Alijani S, Gorbani A, Farahvash T. Estimation of genetic parameters of Wood’s lactation curve parameters using Bayesian and REML methods for milk production trait of Holstein dairy cattle. Journal of Applied Animal Research. 2022;50(1):363–8. doi: 10.1080/09712119.2022.2080211

[37] de Oliveira HR, e Silva FF, Barbosa da Silva MVG, Barbosa Dias de Siqueira OHG, Machado MA, Carmo Panetto JCd, et al. Bayesian Models combining Legendre and B-spline polynomials for genetic analysis of multiple lactations in Gyr cattle. Livestock Science. 2017;201:78–84. doi: 10.1016/j.livsci.2017.05.007

[38] Colonia SRR, Oliveira AdC, Pilonetto F, Dauria BD, Mourão GB, Machado PF, et al. Genetic parameters for milk yield, casein percentage, subclinical mastitis incidence and sexual precocity using Bayesian linear and threshold models. Animal Production Science. 2022;62(8):792–801. doi: 10.1071/AN20313

[39] Azevedo Junior J, Gonçalves TdM, de Souza JC, Rodriguez MAP, Costa CN, Carvalheira JGV Adjustment of lactation curves of Holstein cows from herds of Minas Gerais, Brazil. 2018;10.5539/jas.v10n2p1. doi: 10.5539/jas.v10n2p1

[40] Ali I, Muhammad Suhail S, Shafiq M. Heritability estimates and genetic correlations of various production and reproductive traits of different grades of dairy cattle reared under subtropical condition. Reproduction in Domestic Animals. 2019;54(7):1026–33. doi: 10.1111/rda.13458 31077461

[41] Moncur VS, Hardie LC, Dechow CD. Genetic analysis of daily milk yield variability in Holstein dairy cattle in an experimental herd. Livestock Science. 2021;244:104397. doi: 10.1016/j.livsci.2021.104397

[42] Samaraweera AM, Boerner V, Cyril HW, van der Werf J, Hermesch S. Genetic parameters for milk yield in imported Jersey and Jersey-Friesian cows using daily milk records in Sri Lanka. Asian-Australas J Anim Sci. 2020;33(11):1741–54. Epub 2020/02/29. doi: 10.5713/ajas.19.0798 ; PubMed Central PMCID: PMC7649081. 32106654 PMC7649081

[43] Hernandez-Rivera JA, Molina-Ochoa J, Garcia-Marquez LJ, Prado-Rebolledo OF, Macedo-Barragan RJ, Garcia-Casillas AC, et al. Crossbred Dairy Cattle is the Answer to Improve Environment Dependent Productive and Physiological Responses—A Review. Pakistan Journal of Zoology. 2019;51:773. https://link.gale.com/apps/doc/A586359583/AONE?u=anon~b0b8a0e&sid=googleScholar&xid=3a5c25de.

[44] Otto PI, Guimarães SEF, Calus MPL, Vandenplas J, Machado MA, Panetto JCC, et al. Single-step genome-wide association studies (GWAS) and post-GWAS analyses to identify genomic regions and candidate genes for milk yield in Brazilian Girolando cattle. Journal of Dairy Science. 2020;103(11):10347–60. doi: 10.3168/jds.2019-17890 32896396

[45] Barbero MMD, Fort NM, Schultz ÉB, Melo ALPd, Moura AM. Estimation of genetic parameters in dairy production in girolando cattle. Ciência Animal Brasileira. 2022;23. doi: 10.1590/1809-6891v23e-72300E

[46] Canaza-Cayo AW, Lopes PS, Cobuci JA, Martins MF, Silva MVGBd Genetic parameters of milk production and reproduction traits of Girolando cattle in Brazil. Italian Journal of Animal Science. 2018;17(1):22–30. doi: 10.1080/1828051X.2017.1335180

[47] Barzehkar R, Emamjomeh Kashan N, Asadi Fozi M, Chamani M. A Study on the Association of Days Open with the Genetic Ranking of Iranian Holstein Bulls for the Trait of Milk Yield. Journal of Agricultural Science and Technology. 2023;25(2):331–9. doi: 10.52547/jast.25.2.331

[48] Wahinya PK, Jeyaruban G, Swan A, Magothe T. Estimation of genetic parameters for milk and fertility traits within and between low, medium and high dairy production systems in Kenya to account for genotype-by-environment interaction. Journal of Animal Breeding and Genetics. 2020;137(5):495–509. doi: 10.1111/jbg.12473 32170818

[49] Tlabela MN. Heterogeneity of variance for milk production traits between the low and high input dairy production systems of South Africa 2020.

[50] Boldman KG, Freeman AE. Adjustment for Heterogeneity of Variances by Herd Production Level in Dairy Cow and Sire Evaluation1. Journal of Dairy Science. 1990;73(2):503–12. doi: 10.3168/jds.S0022-0302(90)78698-5

[51] Hagiya K. Development of genetic evaluation for milk production traits of Holsteins in Japan. Animal Science Journal. 2019;90(4):457–61. doi: 10.1111/asj.13190 30763985 PMC6594172

[52] Mulder HA, Veerkamp RF, Ducro BJ, van Arendonk JAM, Bijma P. Optimization of Dairy Cattle Breeding Programs for Different Environments with Genotype by Environment Interaction. Journal of Dairy Science. 2006;89(5):1740–52. doi: 10.3168/jds.S0022-0302(06)72242-1 16606745

[53] Slagboom M, Sørensen AC, Thomasen JR, Liu H, Kargo M, Hjortø L. Ignoring genotype by environment interaction in the genetic evaluation of dairy cattle reduces accuracy but may increase selection intensity. Journal of Dairy Science. 2021;104(12):12756–64. doi: 10.3168/jds.2021-20876 34600706

[54] Chuma-Alvarez JL, Montaldo HH, Lizana C, Olivares ME, Ruiz-López FJ. Genotype × region and genotype × production level interactions in Holstein cows. Animal. 2021;15(9):100320. doi: 10.1016/j.animal.2021.100320 34416556

[55] Behera R, Mandal A, Rai S, Karunakaran M, Mondal M, Ghosh M. Genotype environment interaction on milk production traits of crossbred dairy cows under tropical climatic condition of India. Indian Journal of Animal Research. 2022;56(11):1422–7. doi: 10.18805/IJAR.B-4121

[56] Ducrocq V, Cadet A, Patry C, van der Westhuizen L, van Wyk JB, Neser FWC. Two approaches to account for genotype-by-environment interactions for production traits and age at first calving in South African Holstein cattle. Genetics Selection Evolution. 2022;54(1):43. doi: 10.1186/s12711-022-00735-5 35690732 PMC9188047

[57] Júnior R. N. C. C., Araújo C. V. D., Silva W. C. D., Araújo S. I. D., Lôbo R. B., Nakabashi L. R. M., et al. (2023). Mixed Models in Nonlinear Regression for Description of the Growth of Nelore Cattle. Animals, 13(1), 101. 10.3390/ani13010101 PMC9817709 36611710

[58] Silpa MV, König S, Sejian V, Malik PK, Nair MRR, Fonseca VFC, et al. Climate-Resilient Dairy Cattle Production: Applications of Genomic Tools and Statistical Models. Frontiers in Veterinary Science. 2021;8. doi: 10.3389/fvets.2021.625189 33996959 PMC8117237

[59] Sartori C, Tiezzi F, Guzzo N, Mancin E, Tuliozi B, Mantovani R. Genotype by Environment Interaction and Selection Response for Milk Yield Traits and Conformation in a Local Cattle Breed Using a Reaction Norm Approach. Animals. 2022;12(7):839. doi: 10.3390/ani12070839 35405829 PMC8996846

[60] Vilela D, de Resende J, Leite J, Alves E. The evolution of milk in Brazil in five decades. Revista de Politica Agricola. 2017;26(1):5–24. https://www.cabdirect.org/cabdirect/abstract/20173297908.

[61] Mulim HA, Carneiro PLS, Malhado CHM, Pinto LFB, Mourão GB, Valloto AA, et al. Genotype by environment interaction for fat and protein yields via reaction norms in Holstein cattle of southern Brazil. Journal of Dairy Research. 2021;88(1):16–22. Epub 2021/02/17. doi: 10.1017/S0022029921000029 33593451

[62] Streit M, Reinhardt F, Thaller G, Bennewitz J. Reaction norms and genotype-by-environment interaction in the German Holstein dairy cattle. Journal of Animal Breeding and Genetics. 2012;129(5):380–9. doi: 10.1111/j.1439-0388.2012.00999.x 22963359

[63] Pfeiffer C, Fuerst C, Schwarzenbacher H, Fuerst-Waltl B. Genotype by environment interaction in organic and conventional production systems and their consequences for breeding objectives in Austrian Fleckvieh cattle. Livestock Science. 2016;185:50–5. 10.1016/j.livsci.2016.01.011.

