# Supplementary material for: Heterogeneity of variance and genetic parameters for milk production in cattle, using Bayesian inference
Source: PLoS One. 2023 Jul 12;18(7):e0288257. doi: 10.1371/journal.pone.0288257 (PMC10337916; doi:10.1371/journal.pone.0288257)
Supplement: S1 File — (PDF) [file pone.0288257.s001.pdf]

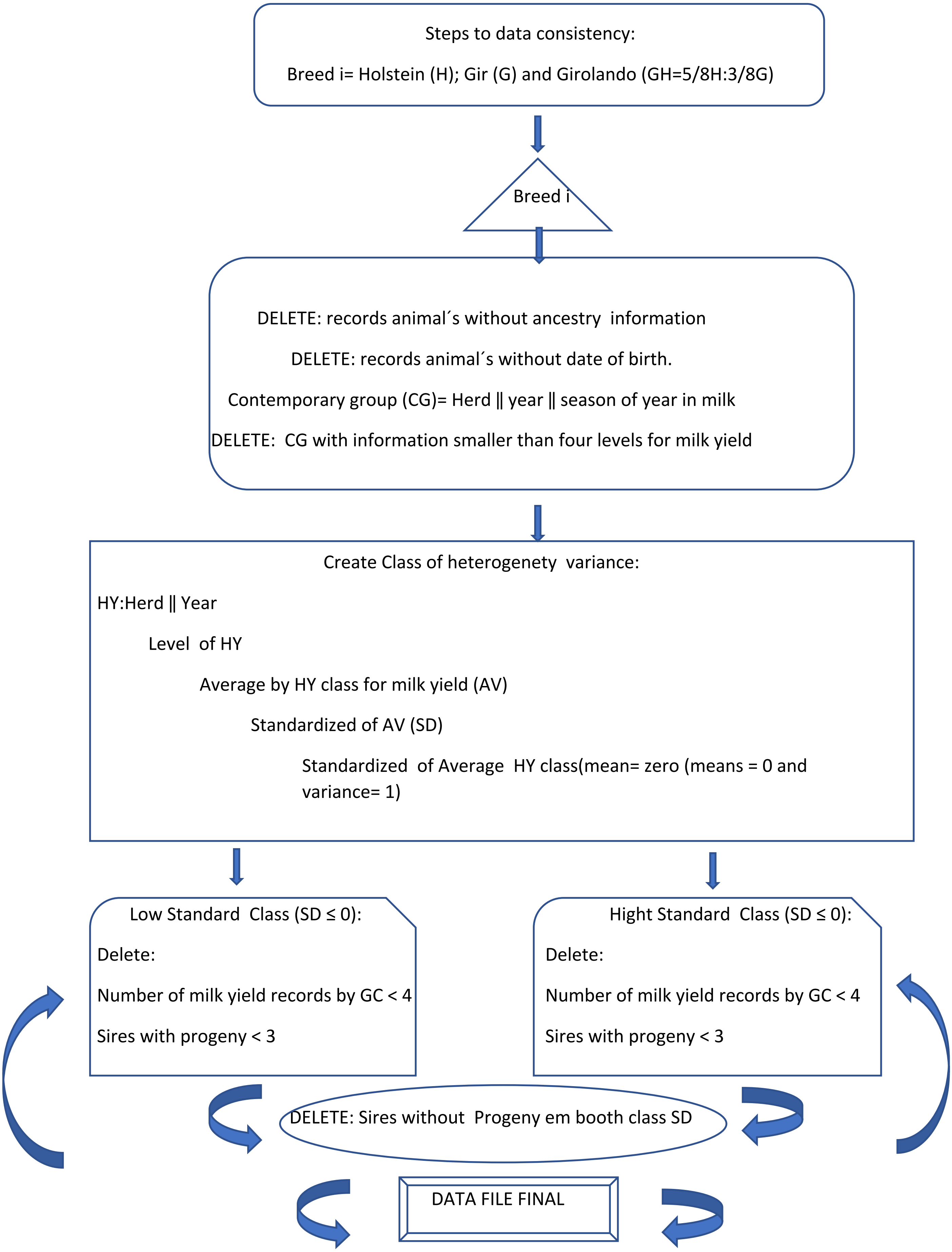
Steps to data consistency:
Breed i= Holstein (H); Gir (G) and Girolando (GH=5/8H:3/8G)
Breed i
DELETE: records animal´s without ancestry information
DELETE: records animal´s without date of birth.
Contemporary group (CG)= Herd || year || season of year in milk
DELETE: CG with information smaller than four levels for milk yield
Create Class of heterogenety variance:
HY:Herd || Year
Level of HY
Average by HY class for milk yield (AV)
Standardized of AV (SD)
Standardized of Average HY class(mean= zero (means = 0 and variance= 1)
Low Standard Class (SD ≤ 0):
Delete:
Number of milk yield records by GC < 4
Sires with progeny < 3
Hight Standard Class (SD ≤ 0):
Delete:
Number of milk yield records by GC < 4
Sires with progeny < 3
DELETE: Sires without Progeny em booth class SD
DATA FILE FINAL
